# Multiple Myeloma Presenting With Sudden Paraplegia

**DOI:** 10.7759/cureus.86415

**Published:** 2025-06-20

**Authors:** Hugo Veiga, Manuel Almeida, Pedro Duarte, José Artur Paiva, Elisabete Monteiro

**Affiliations:** 1 Intensive Care Department, Hospital de São João, Porto, PRT; 2 Intensive Care Department, Hospital de Portimão, Portimão, PRT; 3 Intensive Care Department, Hospital Divino Espírito Santo, Ponta Delgada, PRT; 4 Surgery and Physiology Department, Faculty of Medicine, University of Porto, Porto, PRT; 5 Intensive Care Medicine Department, Hospital de São João, Porto, PRT

**Keywords:** dorsal spinal mass, extra-medullary plasmacytoma, medullary syndrome, multiple myeloma, paraplegia

## Abstract

Multiple myeloma (MM) is a malignant neoplasia predominantly characterized by systemic manifestations, but atypical presentations can challenge diagnosis and management, as in the case of solitary plasmacytoma. We present a case of a 60-year-old woman with sudden paraplegia due to a solitary dorsal spinal mass compressing the spinal cord. Emergent surgical decompression prevented further neurological deterioration, but permanent paraplegia remained. This case underlines the importance of recognizing atypical presentations of multiple myeloma, such as solitary spinal lesions, which may mimic other conditions. Early diagnosis and intervention are crucial to prevent irreversible neurological sequelae. Prompt surgical decompression, followed by systemic therapy, can optimize outcomes in patients with this pathology. Multidisciplinary collaboration and aggressive management of complications, as well as early rehabilitation, are also essential to address the complex needs of these patients and improve outcomes.

## Introduction

Multiple myeloma (MM) is a neoplastic disease characterized by clonal plasma cell proliferation, with well-defined diagnostic criteria, that typically presents with the known CRAB features (which stands for hyperCalcemia, Renal failure, Anemia, and Bone lesions) [1]. A solitary plasmacytoma is a tumor lesion composed of clonal plasma cells, which are histologically identical to those seen in MM, but without systemic involvement. However, sometimes, MM can present as a single lesion with no other symptoms, a plasmacytoma, but with underlying bone marrow infiltration by clonal plasma cells. Here, we report a case of this atypical presentation, with a single mass that had grown silently in the dorsal spine until the compression of the spinal cord resulted in sudden paraplegia. Our aim is to report this atypical and seldom-seen presentation and discuss the impact of early intervention on the functional neurological outcome.

## Case presentation

A 60-year-old woman, with a medical history of heavy smoking, asthma-chronic obstructive pulmonary disease (COPD) overlap syndrome, Child-Pugh A alcoholic liver cirrhosis, type 2 diabetes mellitus treated with insulin, and arterial hypertension. She was on lisinopril, atorvastatin, fenofibrate, and clopidogrel (as primary prevention).

She presented at the emergency department (ED) with a one-day history of dorsal pain, with paraplegia and numbness of the lower limbs, besides vesical and rectal incontinence.

At the ED, she presented with a Glasgow Coma Scale of 15 points and no neurological deficits concerning cranial nerves or upper limbs. However, she had paraplegia with strength grade 0/5 in all segments of the lower limbs and absent sensitivity below the T10 level.

**Figure 1:**
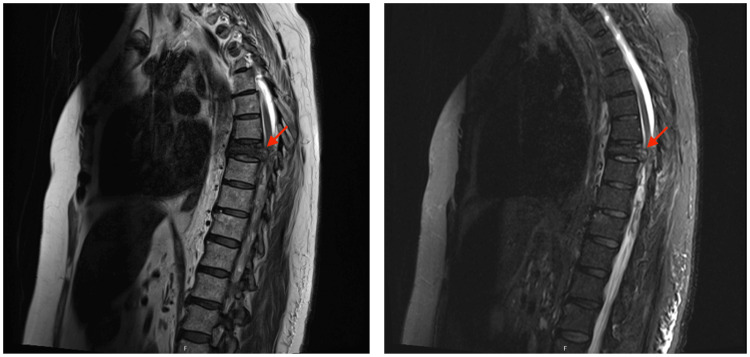
Magnetic resonance imaging (MRI) of the dorsal spine showing an expansive lesion, centered on the T8 vertebral body, causing the compression of the spinal cord (red arrows).

Emergent computed tomography (CT) and magnetic resonance imaging (MRI) of the spinal cord were performed on the patient, which showed an expansive lesion centered on the T8 vertebral body, with extension to the pedicles, transverse processes, and lateral masses of the respective vertebral piece (Figure 1). It was accompanied by the marked thickening of the anterolateral paravertebral soft tissues, as well as edema and the enhancement of the posterolateral paravertebral soft tissues, with craniocaudal extension between T6 and T11. The changes described resulted in the clear compression of the medullary cord, particularly significant at the level of T8, where T2/short tau inversion recovery (STIR) medullary hypersignal was evident, indicating myelopathy/medullary "suffering." These findings were suggestive of a lesion of probable neoplastic nature, either primary or secondary.

**Figure 2:**
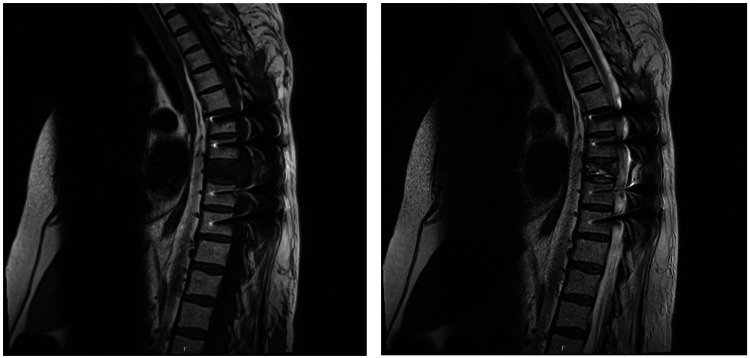
Magnetic resonance imaging (MRI) of the dorsal spine after surgery for the decompression of the spinal cord, exhibiting the removal of the tumor lesion along with part of T8 and the fixation of the remaining spine.

She underwent urgent surgery for spinal decompression, with laminectomy of T8, the removal of the tumor lesion, and the fixation of the remaining spine (Figure 2). The lesion was sent for anatomopathological analysis. She was admitted to the intensive care unit (ICU) after the procedure.

There were no intra- or perioperative complications. Regarding the neurological deficits, she recovered some sensitivity in both lower limbs (although maintaining paresthesias) and some movement of the toes, in the following days. However, she maintained plegia of the remaining lower limbs, especially in the big proximal muscles.

**Figure 3:**
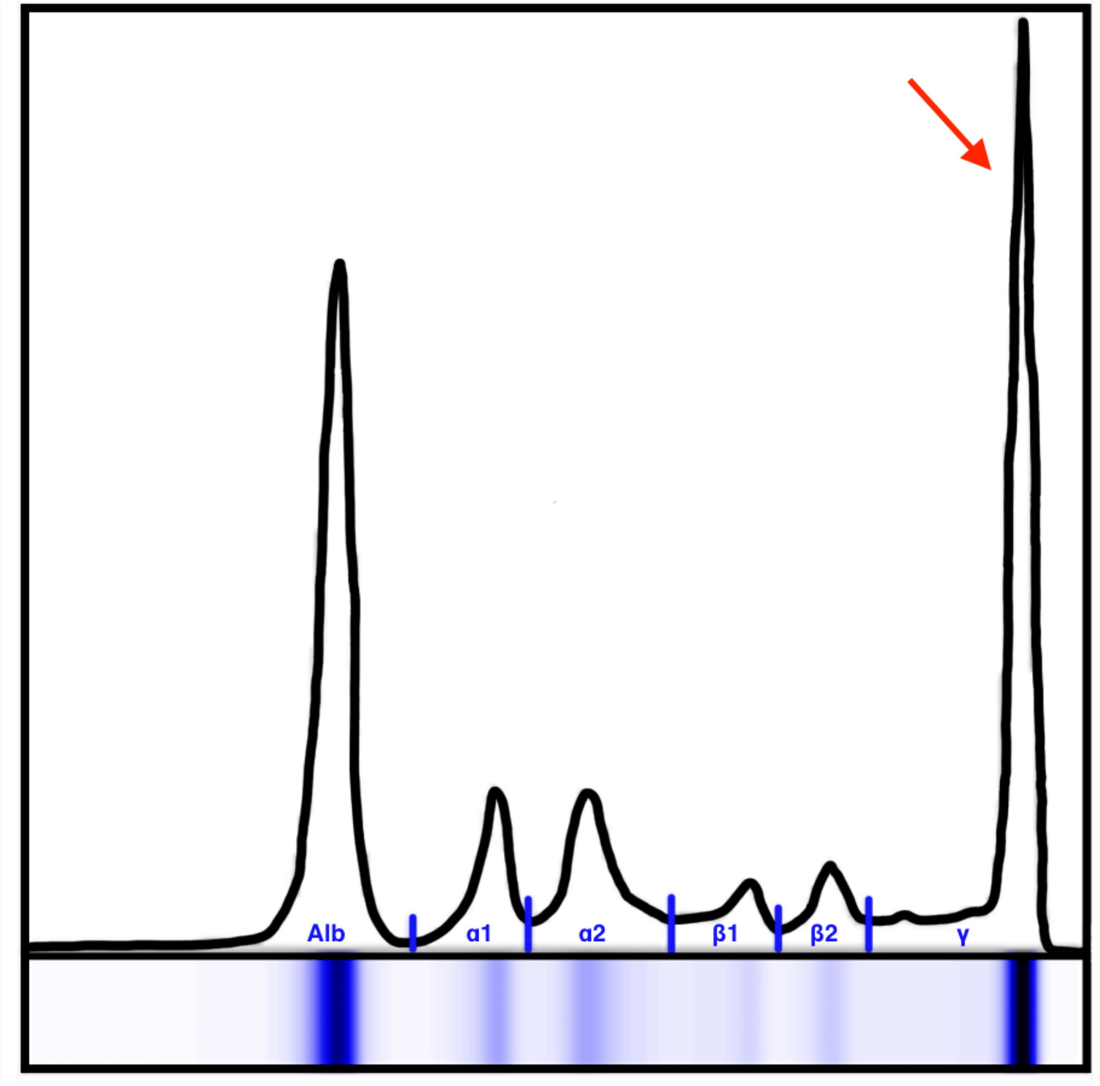
Serum protein electrophoresis (SPEP) exhibiting a peak corresponding to a monoclonal proliferation in the gamma (γ) region (red arrow). The other visible peaks correspond to normal curves in the albumin, alpha-1, alpha-2, beta-1, and beta-2 regions.

The extemporaneous anatomopathological examination of the spine lesion during surgery was inconclusive, but given the suspicion of neoplasm, we started the diagnostic evaluation aiming for the most probable causes. A body CT was performed, as well as an MRI of the whole spine. No other lesions were found. Meanwhile, preliminary results of the histology of the lesion were suggestive of a plasmacytoma. She did not have anemia or hypercalcemia, and kidney function was normal. Serum protein electrophoresis (SPEP) revealed a peak in the gamma region (Figure 3), with an increase in IgG and kappa light chains in the immunofixation. The 24-hour urine protein electrophoresis (UPEP) and urine immunofixation were normal. The bone marrow aspirate of the sternum was performed and revealed ~30% monoclonal plasma cells. A diagnosis of MM was made, and bortezomib was started in association with high-dose dexamethasone. The immunophenotyping of the bone marrow showed an anomalous population compatible with MM. Regarding risk stratification, beta-2 microglobulin was 3.6 mg/L, serum albumin was 2.8 g/dL, and lactate dehydrogenase (LDH) was 235 U/L, which fits stage II in the Revised International Staging System (RISS). Definitive anatomopathological examination also confirmed morphological and phenotypic characteristics compatible with plasmacytoma/multiple myeloma IgG/kappa.

During ICU stay, COPD worsened due to a respiratory infection, requiring sedation and ventilation for seven days. Later, she had a surgical wound infection with no microbiological isolation, requiring 10 days of empirical antibiotic therapy. She was transferred after 41 days to the hematology ward. She also started early motor rehabilitation at day 1 of admission and was only interrupted by the need for re-sedation, even though, at the time of transfer, the neurological deficits remained unchanged.

## Discussion

This case illustrates a presentation of a plasmacytoma, found as an isolated mass in the dorsal spine, causing neurological deficits and leading to paraplegia, as a manifestation of MM.

Epidural spinal cord compression (ESCC) is the second most common neurological complication in cancer patients, and it is an oncological emergency. Early diagnosis and intervention may prevent debilitating neurological sequelae including paraplegia and incontinence [2]. The degree of spinal cord compression influences the urgency of therapy and can be described by the ESCC scale, a valid and reliable instrument based on T2-weighted magnetic resonance images that accounts for recent advances in the treatment of spinal metastases [3]. Magnetic resonance imaging (MRI) is considered the gold standard imaging for the differentiation between pathological fractures associated with metastatic lesions and benign fractures [4]. Any patient with lower-extremity weakness, bowel or bladder incontinence, clonus, and down-going toes on the plantar reflex examination or whose sphincter is patulous must be evaluated and treated immediately [5]. Patients who present with spinal pain but no neurological compromise should have an MRI scan performed with STIR and T1-weighted images to detect any spinal fractures. The T1-weighted images may be better than the STIR images in highlighting the fracture line in vertebrae infiltrated with a myelomatous deposit. Those that present with neurological compromise may have spinal cord, cauda equina, or nerve root compression. It is imperative, in patients with neurological compromise, to get not only an MRI scan with STIR and T1-weighted images but also a CT scan (with soft tissue windowing) to delineate whether it is the bone or soft tissue compromising the neurological structure [6].

The early recognition of spinal cord compression is crucial, as delayed intervention can lead to irreversible neurological deficits.

Surgery may sometimes be required for patients with structural instability of the bone, retropulsed bone, or rapidly progressive symptoms from cord compression, as occurred in our case. Prompt diagnosis and emergent surgical decompression probably prevented further neurological deterioration. However, despite successful decompression, the patient experienced persistent paraplegia, highlighting that time could not be the only factor determining the outcome of these patients. In fact, spinal cord ischemia caused by compression due to a mass can become irreversible depending on several factors such as the severity of compression, the individual's overall health, and specific characteristics of the mass and not only depending on time. Nonetheless, irreversible damage to the spinal cord can occur relatively quickly, within hours, if blood flow is significantly compromised [7].

In all patients presenting with a plasmacytoma, MM should be suspected. Diagnostic evaluation for suspected MM includes imaging studies, serum and urine protein electrophoresis with immunofixation, and bone marrow biopsy. In this case, the diagnosis of MM was based on bone marrow findings and abnormal protein electrophoresis [1,8].

In patients with solitary plasmacytoma of the bone alone (that does not meet the criteria for MM), the primary treatment is localized radiation therapy rather than systemic therapy [9,10]. The use of concurrent, adjuvant, or prophylactic systemic therapy in solitary plasmacytoma is controversial, since some studies have suggested no benefit with systemic therapy [11-13]. However, in our case, despite the initial presentation as a plasmacytoma, the disease met the criteria for the diagnosis of MM, and therefore, systemic treatment for MM was warranted. It may include chemotherapy, immunomodulatory agents, or stem cell transplantation if needed. According to these recommendations, the patient initiated therapy with bortezomib and high-dose dexamethasone, consistent with current treatment guidelines for MM [14].

As with any other neoplastic lesions with ESCC, glucocorticoid therapy is generally considered to be part of the standard regimen for symptomatic ЕSCC, to reduce edema associated with the lesion, as a bridge to definitive treatment or for the palliation of pain [15-17]. This was the case in our patient who immediately started dexamethasone on day 1, even before the indication of dexamethasone as part of the combined regimen for the treatment of MM.

In addition to the typical treatment of the disease, given the presentation of this case as a plasmacytoma compressing the spinal cord, resulting in paraplegia, the early initiation of physical rehabilitation was crucial to maximize functional recovery. Multidisciplinary management involving hematologists, neurosurgeons, intensivists, and physiatrists is essential to optimize outcomes in complex cases such as this.

## Conclusions

This case underscores the importance of recognizing atypical presentations of multiple myeloma (MM), such as solitary plasmacytoma, which can mimic other pathologies, including metastatic tumors or epidural abscesses. Accurate and timely diagnosis is essential, as early intervention may prevent irreversible neurological damage. In patients presenting with spinal cord compression, prompt surgical decompression is often indicated and can significantly improve outcomes. The use of glucocorticoids is also recommended to reduce inflammation and edema. If MM is diagnosed, systemic therapy should be initiated, as the long-term treatment approach for plasmacytoma generally aligns with that of MM. However, when plasmacytoma presents without systemic involvement, initial management may differ. Multidisciplinary collaboration is crucial to address the diagnostic and therapeutic complexities in such cases and to ensure comprehensive patient care.
